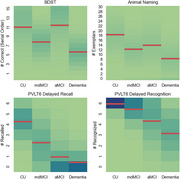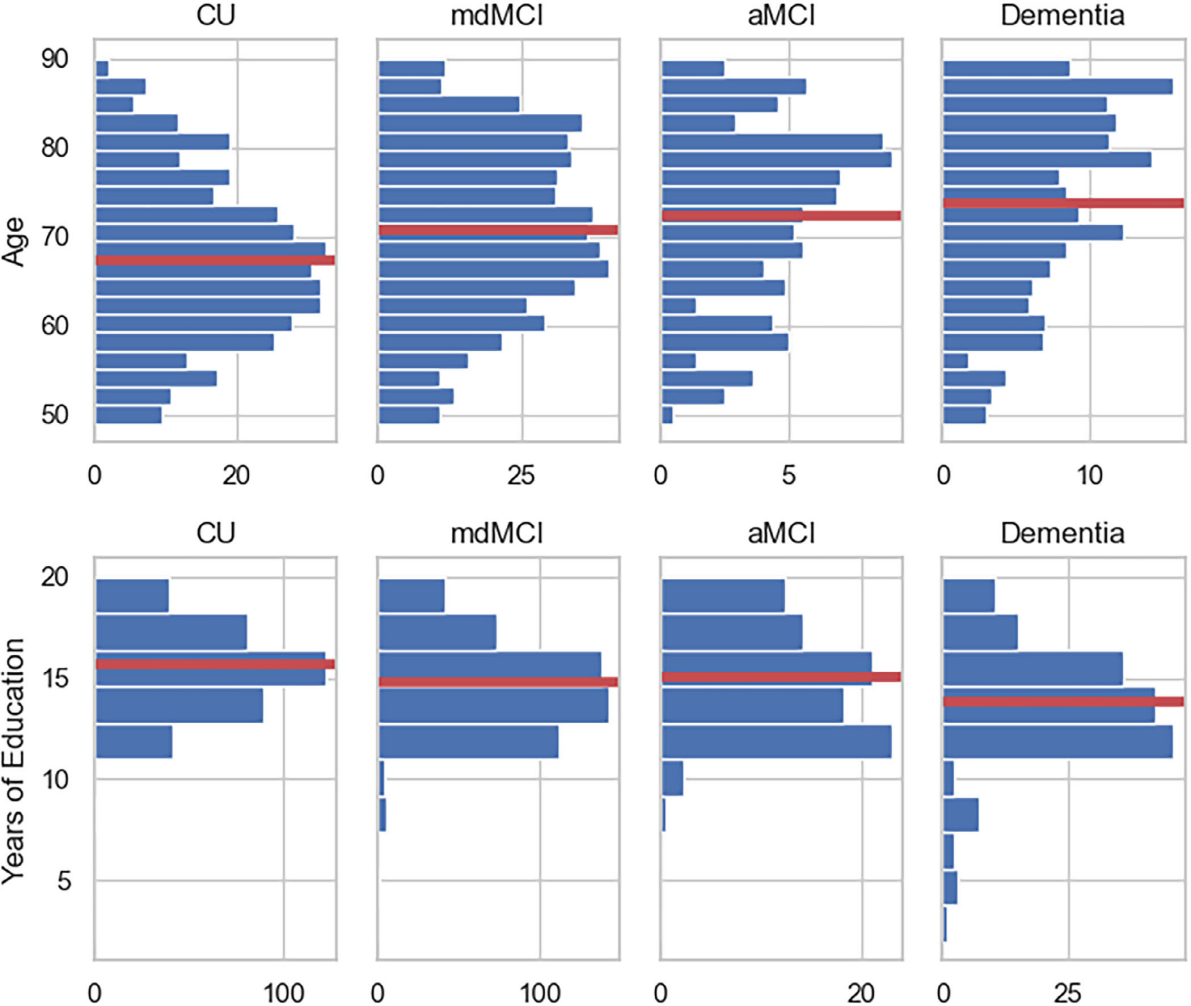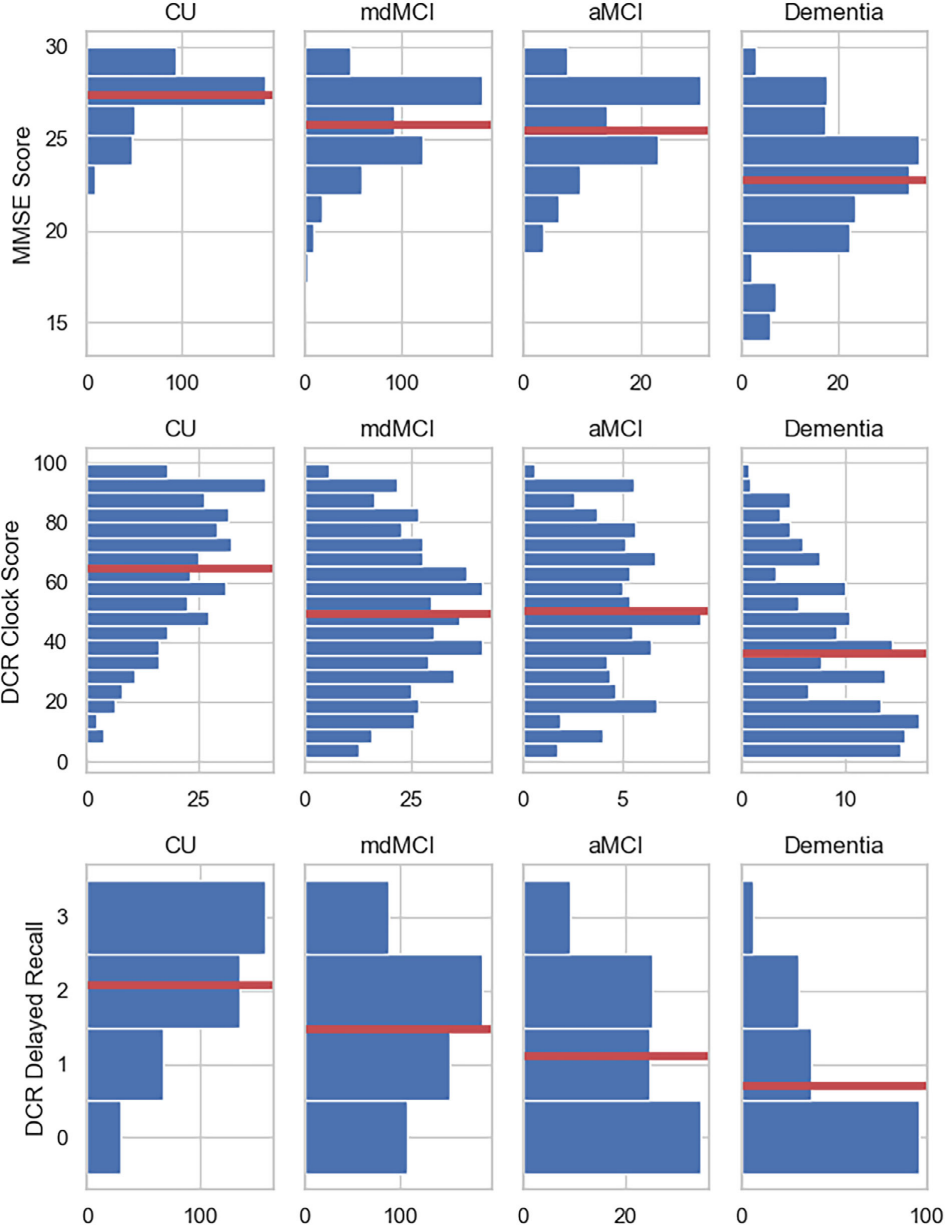# Exploratory Semi‐Supervised Clustering to Identify Dementia and MCI Cohorts with a Brief Automated Digital Assessment Protocol

**DOI:** 10.1002/alz70857_103730

**Published:** 2025-12-24

**Authors:** Daniel J Schulman, David J. Libon, Ali Jannati, Rodney Swenson, Sean Tobyne

**Affiliations:** ^1^ Linus Health, Boston, MA, USA; ^2^ Rowan University, Glassboro, NJ, USA; ^3^ Rowan‐Virtua School of Osteopathic Medicine, New Jersey Institute for Successful Aging, Stratford, NJ, USA; ^4^ University of North Dakota School of Medicine and Health Sciences, Grand Forks, ND, USA

## Abstract

**Background:**

The Apheleia‐001 study deployed a panel of digital and blood‐based biomarkers designed to reduce the screen failure rate among participants interested in therapeutic clinical trials for Alzheimer's disease. Participants were assessed with the Linus Health Digital Assessment of Cognition (DAC), an automated assessment including the 6‐word Philadelphia (repeatable) Verbal Learning Test (PVLT), the semantic ‘animal’ fluency test, and a five‐digit backward digit span test. We aimed to explore how best to classify participants into cognitively unimpaired (CU), mild cognitive impairment (MCI), and dementia cohorts based on DAC metrics. Because Apheleia‐001 did not establish a gold‐standard diagnosis, we developed a semi‐supervised method that augmented the study data with a smaller dataset of memory clinic patients with a clinical diagnosis based on a lengthy and traditional neuropsychological examination.

**Method:**

We combined a dataset (*n* = 1189) from the Apheleia‐001 study with our labeled memory‐clinic dataset (*n* = 106) before fitting a set of modified finite mixture clustering models to DAC scores. Models varied from 3‐5 clusters, consisting of a CU cluster, a dementia cluster, and 1‐3 MCI clusters, with varied probability models for DAC scores. A final model was chosen by expert consensus after considering statistical measures of model fit and agreement with theory. As preliminary construct validation, we tested between‐cluster differences in age, years of education, and performance on two additional assessments: the Linus Health Digital Clock and Recall (DCR) and the MMSE.

**Result:**

Expert consensus selected a 4‐cluster model that demonstrated good model fit, identifying two MCI clusters presenting with amnestic and mixed/dysexecutive cognitive profiles. There were significant between‐cluster differences, all in hypothesized directions, i.e., more impaired clusters were older, less educated, and performed more poorly on the DCR and MMSE. Importantly, significant differences between the MCI clusters were found only for the DCR delayed free‐recall, where the amnestic group scored lowest.

**Conclusion:**

While further development and validation are needed, we show evidence that the DAC can, with a 7‐minute automated digital assessment, identify cognitively intact, dementia, and two MCI subtypes.